# An Atypical Finding of Peripheral Retinal Ischemia and Neovascularization in Neurofibromatosis Type 1: A Case Report

**DOI:** 10.7759/cureus.75154

**Published:** 2024-12-05

**Authors:** Wael A Alsakran, Nada Alyousef, Abrar A Alhawsawi, Abeer A Aljahdali, Sahar M Elkhamary

**Affiliations:** 1 Ophthalmology Department, King Khaled Eye Specialist Hospital, Riyadh, SAU; 2 Ophthalmology Department, College of Medicine, University of Jeddah, Jeddah, SAU; 3 Ophthalmology Department, College of Medicine, King Abdulaziz University, Jeddah, SAU; 4 Radiology Department, King Khaled Eye Specialist Hospital, Riyadh, SAU

**Keywords:** neovascular glaucoma, neurofibromatosis type 1, ocular abnormality, retinal ischemia, retinal neovascularization

## Abstract

Neurofibromatosis type 1 (NF1) is an autosomal dominant genetic multisystem phakomatosis that can affect the skin, bones, and nervous system. NF1 typically presents with skin lesions, including freckles, café-au-lait macules, plexiform neurofibromas, and bony dysplasia, and is usually accompanied by a family history of the disorder. Ocular manifestations vary, but iris Lisch nodules and optic nerve gliomas are the most common features. However, patients with NF1 may also present with rare ocular findings, such as neovascular glaucoma, astrocytic hamartomas, choroidal nodules, and retinal vascular abnormalities. Here, we report a case of an 18-year-old female patient, born to consanguineous parents, who had never been diagnosed with NF1, nor had any of her family members. The patient presented with unilateral, long-standing vitreous hemorrhage, significant retinal ischemia, and neovascularization. Her examination revealed typical features of NF1, including iris Lisch nodules, café-au-lait spots, and axillary freckling. Magnetic resonance imaging of the brain and orbits, along with dedicated vascular imaging, was performed, and a sectoral laser treatment was applied to the ischemic retina to prevent neovascular glaucoma.

## Introduction

Neurofibromatosis type 1 (NF1) is an autosomal dominant phakomatosis described phenotypically by von Recklinghausen in 1882 [1]. It is characterized by aberrant proliferation in multiple systems, involving organs derived from the neural crest [2]. NF1 is recognized as one of the most prevalent autosomal dominant disorders, with an estimated incidence of 1 in 2,500 births and a prevalence ranging from 1 in 3,000 to 1 in 4,000 individuals [3,4]. This neurocutaneous disorder results from a genetic mutation in the NF1 gene located on the long arm of chromosome 17. The neurofibromin protein encoded by this gene regulates cell growth [2]. Diagnosis is based on typical clinical findings, including skin freckling, café-au-lait macules, plexiform neurofibromas, iris hamartomas, and optic nerve gliomas [1,5]. However, NF1 exhibits significant phenotypic variability, and some patients may also present with atypical systemic and ophthalmic findings. Systemic findings can include macrocephaly, short stature, scoliosis, epilepsy, cognitive impairment, renal artery stenosis, and pheochromocytoma [1]. Conversely, non-typical ophthalmic features may include cataracts, narrow-angle or neovascular glaucoma, retinal astrocytic hamartomas, and choroidal hamartomas [1,6].

In the context of NF1, distinctive vasculopathy may affect up to 10% of patients [7,8]. This vasculopathy can manifest as neovascularization, aneurysms, malformations, or stenosis, frequently affecting large systemic vessels such as the renal, mesenteric, aortic, and carotid circulations [7-9]. In NF1 patients, retinal vascular manifestations typically correspond to retinal capillary hemangiomatosis [10], although there are sporadic, documented cases of retinal vascular occlusive disease [11,12]. We describe a young Saudi woman with NF1 who presented with rare and atypical findings of unilateral peripheral retinal ischemia and retinal vascular abnormalities, which led to secondary retinal neovascularization and vitreous hemorrhage. Early diagnosis of this syndrome and its associated findings is essential for improving visual prognosis, as it enables earlier initiation of appropriate interventions.

## Case presentation

An 18-year-old woman was born to a consanguineous marriage. She had unremarkable medical and surgical histories and denied any prior head trauma or neurological deficits. Additionally, she had never undergone an ocular examination by an ophthalmologist. The patient complained of blurred vision and intermittent floaters in her right eye, which had started a year before the presentation. Upon examination, the best-corrected visual acuity (BCVA) was 20/100 in the right eye and 20/20 in the left eye. The intraocular pressure (IOP) measured 20 mmHg in the right eye and 19 mmHg in the left eye. She exhibited total ocular motility and mild exophoria on ocular alignment, and no tropia or nystagmus was noted during the assessment. Both eyelids appeared normal, with equal palpebral fissures and no mass lesions.

**Figure 1:**
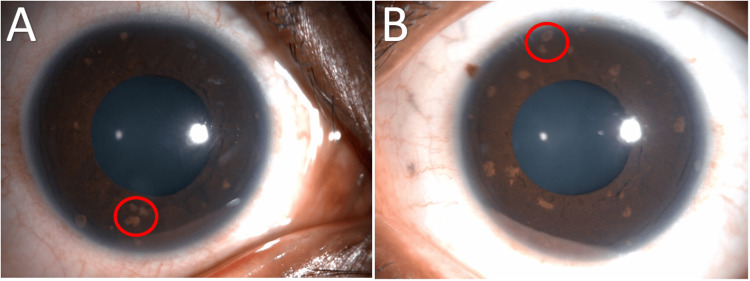
External images External images of the right (A) and left (B) eye show dispersed light-pigmented clumps on the anterior surface of the iris, resembling Lisch nodules (red circles).

**Figure 2:**
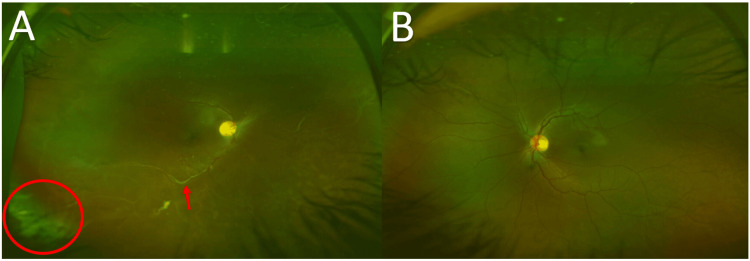
Optos ultra-widefield images (A) The right fundus shows vitreous haze with inferior dehemoglobinized vitreous hemorrhage (red circle) and an ischemic retina nasally and inferiorly with sclerosed blood vessels (red arrow) and telangiectatic changes; (B) The left fundus was within normal limits.

The slit-lamp examination revealed clear anterior segments, along with a remarkable discovery of iris hamartomas, also known as Lisch nodules, in both eyes (Figure 1). There was no neovascularization of the iris (NVI). Fundus examination of the right eye revealed a vitreous haze due to inferior dehemoglobinized vitreous hemorrhage and ischemic peripheral retina, primarily involving the nasal and inferior quadrants. This was accompanied by microvascular abnormalities, including sclerosed and tortuous blood vessels, multiple collateral tufts, and neovascularization elsewhere (NVE) (Figure 2). The left eye fundus examination was unremarkable, showing no peripheral retinal ischemia or NVEs.

**Figure 3:**
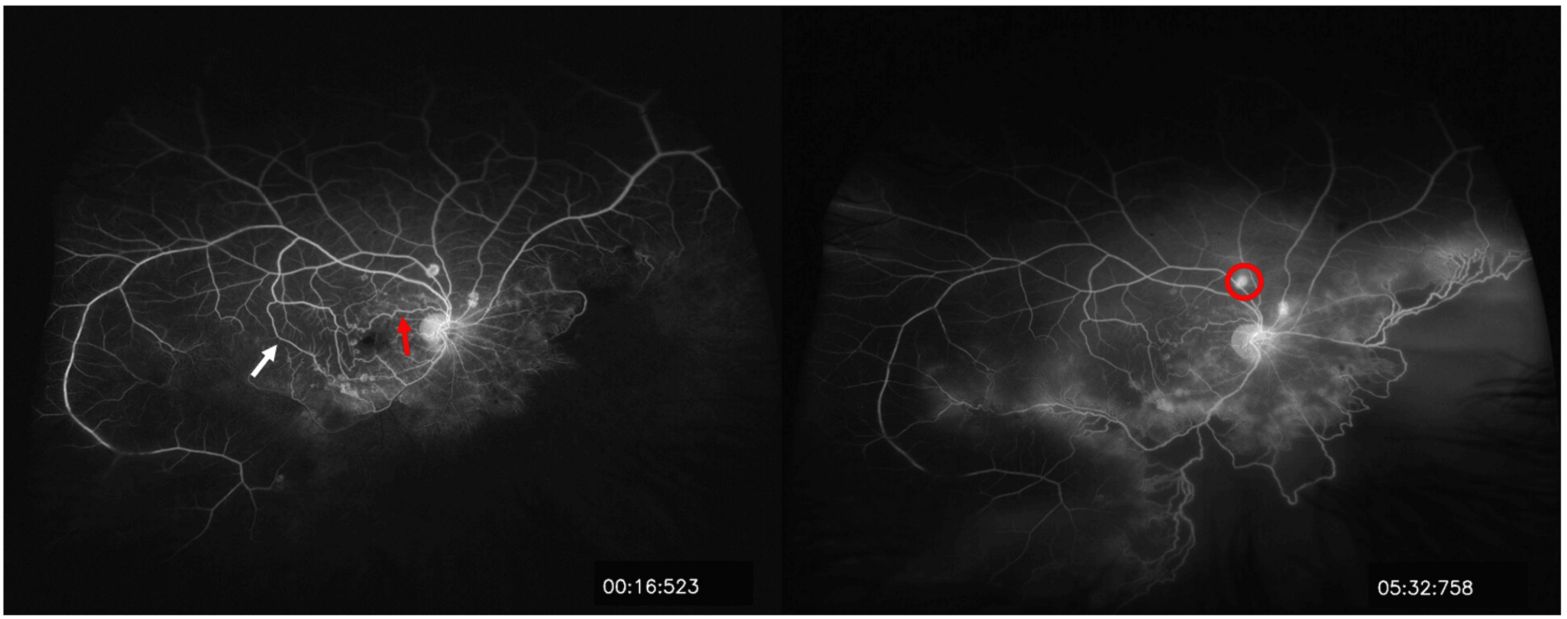
Fundus fluorescein angiography of the right eye Fundus fluorescein angiography of the right eye shows peripheral non-perfusion in the nasal and inferior retina, with telangiectatic and tortuous blood vessels (red arrow), and collaterals between perfused and non-perfused retina (white arrow), associated with multiple NVEs that leak in the late recirculation phase (red circle). NVE, neovascularization elsewhere

**Figure 4:**
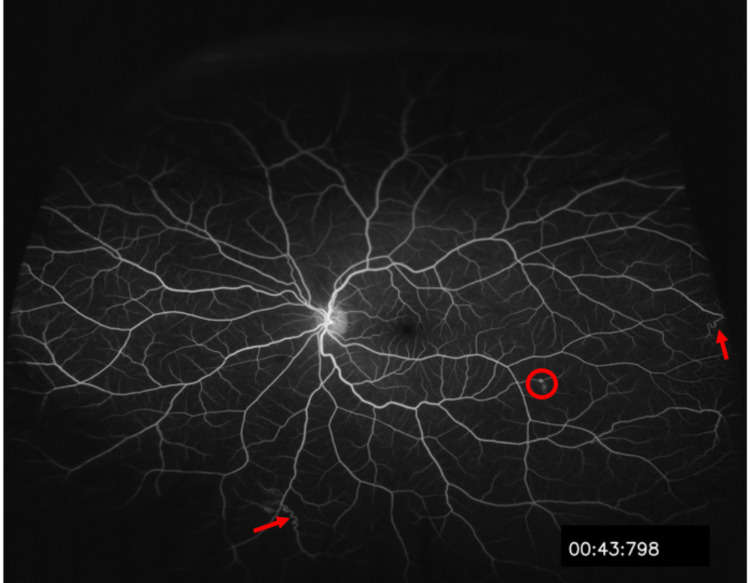
Fundus fluorescein angiography of the left eye Fundus fluorescein angiography of the left eye shows normal perfusion, with no retinal ischemia or neovascularization - spiraling and tortuous configurations involving some of the vessels, both at the posterior pole and at the far periphery (red arrows), in addition to a cork-screw appearance near a major vessel (red circle).

Fluorescein angiography (FA) of the right eye demonstrated peripheral non-perfusion in the nasal and inferior retinas, with telangiectatic and looping blood vessels connecting the perfused and non-perfused areas. Leakage was observed from multiple NVEs, as well as from collaterals in the macular region (Figure 3). FA of the left eye showed tortuous configurations of some vessels at both the posterior pole and the far temporal periphery, including a corkscrew vascular abnormality near a significant major vessel that was not apparent on examination (Figure 4).

**Figure 5:**
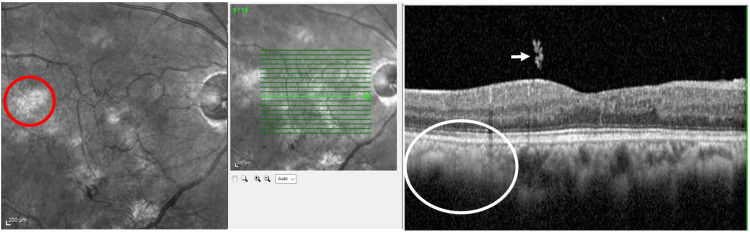
Optical coherence tomography (OCT) with the corresponding en-face image of the right eye Optical coherence tomography (OCT) with the corresponding en-face image of the right eye shows choroidal hamartomas as bright, patchy spots (red circle), with the corresponding OCT through this patch showing hyper-reflective choroid obscuring the reflectivity of some of the choroidal vessels (white circle). OCT also shows a tuft of hyper-reflectivity at the vitreous, corresponding to the abnormal vasculature (white arrow), in addition to disorganized retinal inner layers.

**Figure 6:**
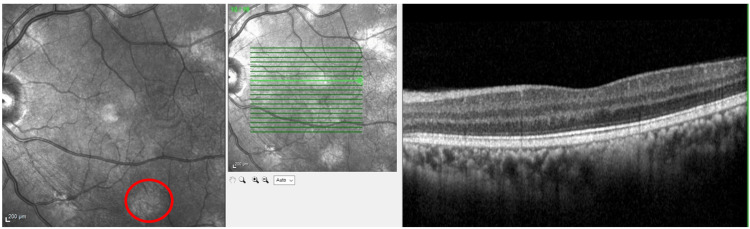
Optical coherence tomography (OCT) with the corresponding en-face image of the left eye The en-face optical coherence tomography (OCT) image of the left eye shows bright, patchy spots in the posterior pole (red circle), with the corresponding OCT showing a normal choroid, as the arrow doesn’t cross through any of these choroidal patches, and normal retinal stratification.

**Figure 7:**
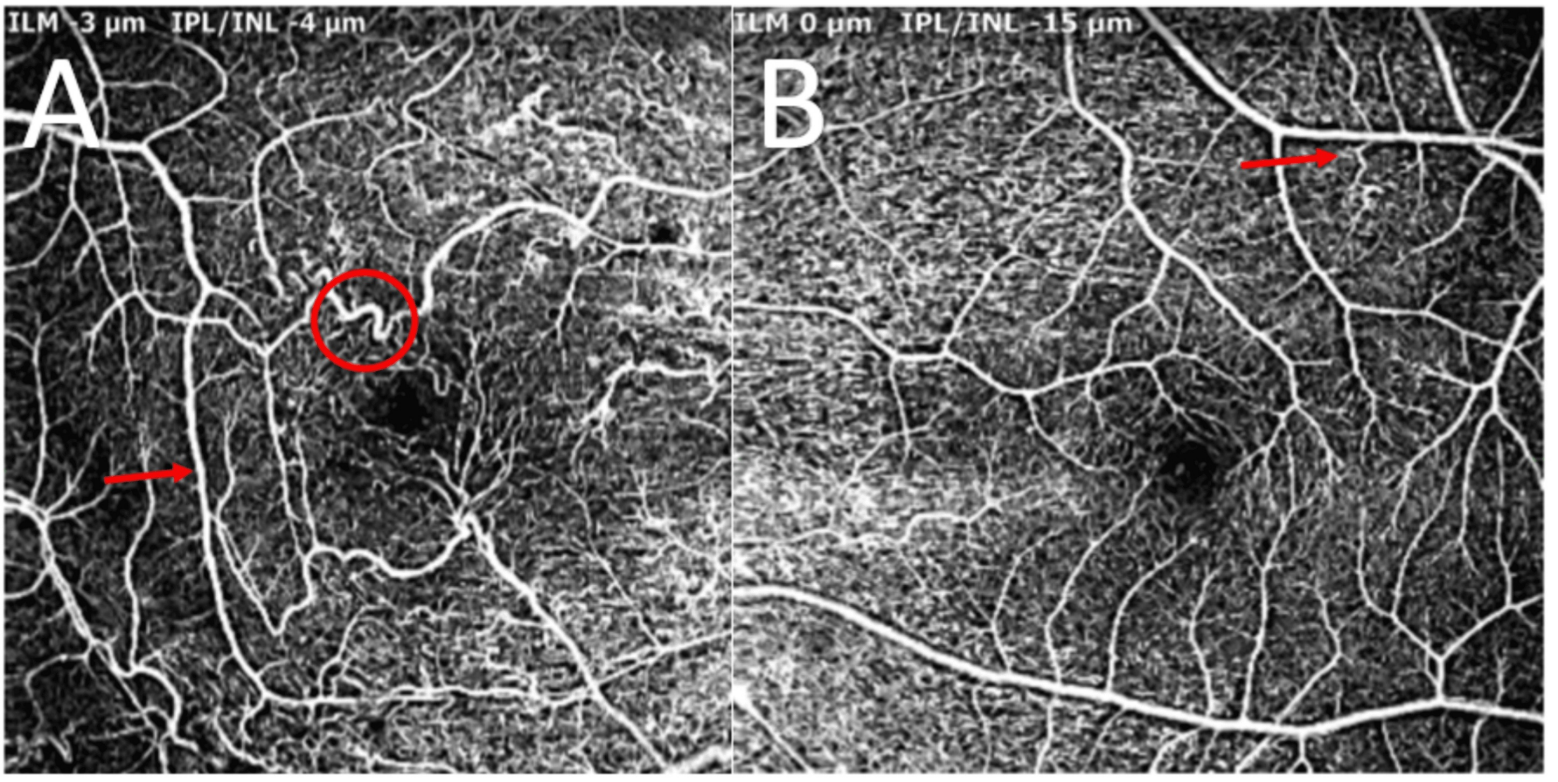
Optical coherence tomography angiography (OCTA) images Optical coherence tomography angiography (OCTA) of the right eye (A) shows multiple vascular abnormalities in the superficial vascular plexus, including vascular tortuosity (red circle) and abnormal communication in a collateral configuration (red arrow). The left eye (B) shows simple vascular tortuosity (red arrow), involving the superficial vascular plexus at the superior arcade.

Macular spectral-domain optical coherence tomography (SD-OCT) (Spectralis, Heidelberg Engineering, Heidelberg, Germany) of the right eye showed regular retinal stratification, with hyperreflective epiretinal lesions extending into the vitreous, indicative of NVEs. Choroidal nodules were identified in both eyes, appearing as hyperreflective signal patches in the choroidal layer, devoid of surrounding blood vessels. On en-face OCT images, these nodules appeared as bright patches (Figures 5-6). Optical coherence tomography angiography (OCTA) revealed areas of tortuous and spiraled blood vessels in the superficial capillary plexus, representing microvascular abnormalities, alongside areas lacking flow signals, indicative of ischemic regions (Figure 7).

Based on the examination findings of nodules in the iris and choroid, a presumptive diagnosis of NF1 was made. This diagnosis was confirmed following skin examinations, which revealed numerous café-au-lait spots and axillary freckling. Two family members were examined and showed no findings suggesting NF1, indicating that the patient’s disorder resulted from a de novo mutation. The right eye underwent urgent sectoral retinal photocoagulation due to posterior segment ischemia and neovascularization, but she declined an intravitreal injection of anti-vascular endothelial growth factor.

**Figure 8:**
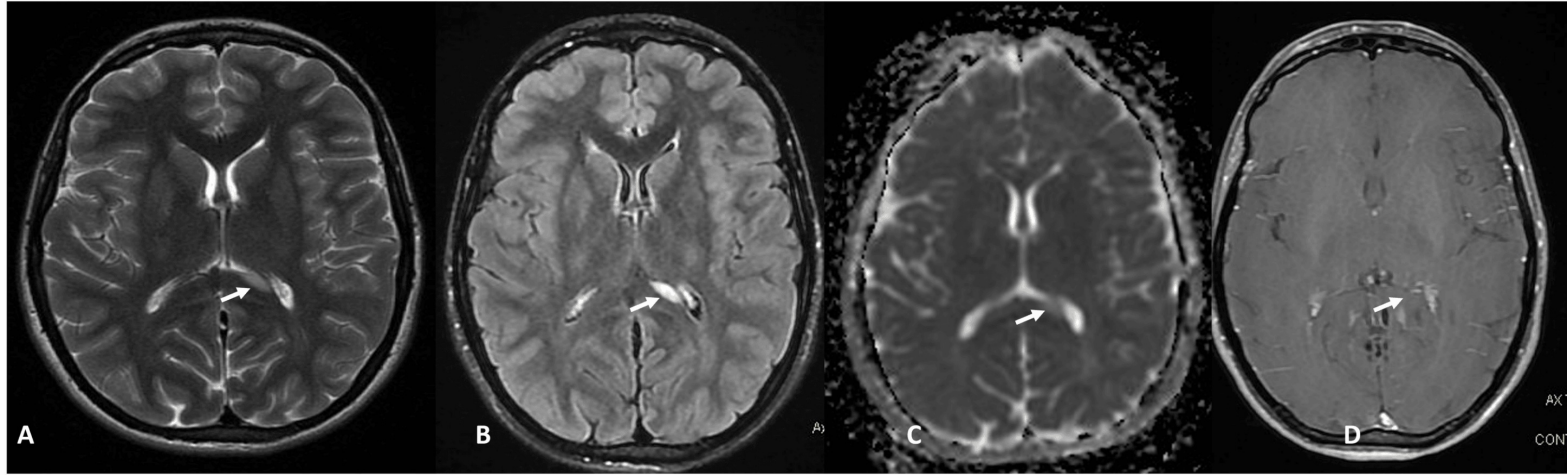
Different sequences axial magnetic resonance images (A) T2WI shows a discrete high-signal lesion (white arrow) on T2-weighted images involving the left side of the splenium of the corpus callosum, more clearly seen on FLAIR sequences (B), with no restricted pattern on diffusion-weighted imaging (ADC) (C), and no enhancement on post-contrast study (D). ADC, apparent diffusion coefficient; FLAIR, fluid-attenuated inversion recovery; T2WI, T2-weighted imaging

We arranged urgent radiographic imaging, including carotid Doppler ultrasound, contrast-enhanced magnetic resonance imaging (MRI) of the brain and orbits, and magnetic resonance angiography (MRA) and magnetic resonance venography (MRV). The vascular imaging was unremarkable, with no significant stenosis or occlusion. The MRI did not reveal an optic pathway glioma but did show a single non-enhancing lesion on the left side of the corpus callosum splenium, touching the ependymal surface in a lentiform-like pattern - a finding that can be observed in some patients with NF1 (Figure 8).

**Figure 9:**
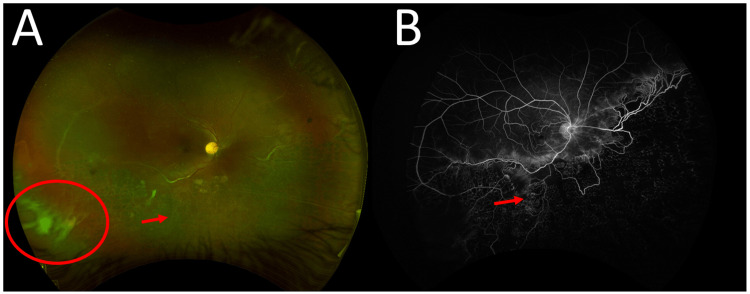
Post-treatment images Post-treatment Optos ultra-widefield image (A) and fundus fluorescein angiography (B) of the right eye show persistent inferior dehemoglobinized vitreous hemorrhage (red circle), with scars of laser treatment (red arrows) and the absence of new-onset neovascularization.

In addition to imaging, laboratory investigations were conducted to rule out other etiologies of ischemia, including glycosylated hemoglobin, lipid profile, hemoglobin electrophoresis, coagulation profile, and inflammatory and infectious panels. All laboratory results were unremarkable, leading to the identification of atypical posterior segment ischemia and neovascularization due to NF1 ocular vasculopathy. During subsequent visits, the patient remained stable, with regression of neovascularization and no development of ocular neovascularization or neovascular glaucoma (Figure 9). She also participated in a multidisciplinary approach and was assessed by the internal medicine team for regular general evaluations, including routine repeat structural and vascular imaging.

## Discussion

Systemic vascular occlusion associated with NF1 has been reported and may involve the mesenteric, cerebral, renal, aortic, and celiac vessels [7-9]. “NF1 vasculopathy” refers to aneurysms, stenoses, and arteriovenous malformations occurring in individuals with NF1, as medical literature notes [7-9]. The pathophysiology of vascular occlusion is linked to the growth and expansion of Schwann cells, as well as the dysplasia of smooth muscle cells in the arteries. This results in a gradual narrowing of the vessel and subsequent occlusion, with secondary fibrous alterations [9]. The NF1 gene functions as a tumor suppressor, and mutations in this gene lead to endothelial and pericyte cell proliferation, which increases the angiogenic response to ischemia [13]. Furthermore, decreased neurofibromin protein expression in endothelial cells may impact interactions between these cells and pericytes, resulting in aberrant cell proliferation in both cell types and eventual vascular occlusion [13].

Numerous reports have described retinal vascular alterations of varying degrees in NF1 [14]. Three distinct vascular abnormalities have been identified in the literature: simple vascular tortuosity, corkscrew retinal vessel configuration, and a moyamoya-like pattern [8,14]. Corkscrew retinal vessels exhibit a more spiral configuration than simple vascular tortuosity, while tortuous vessels ending in a “puff of smoke” indicate a moyamoya-like appearance. Microvascular abnormalities were found in 31.4% of NF1 patients in a large cohort study [8]. Among those patients, simple vascular tortuosity was observed in 74.3%, with 42.8% displaying the corkscrew pattern and 15.2% showing the moyamoya-like type [8]. The exact pathogenesis of these retinal vascular alterations is not well understood. However, they appear to align with the pathophysiology of systemic vascular abnormalities [8]. Studies have indicated that aberrant neurofibromin may lead to a loss of integrity in the endothelial cell layer, potentially allowing vascular smooth muscle cells to increase [9].

Furthermore, endothelial cells and pericytes interact differently when neurofibromin expression is lost, providing both cell types with signals to increase [13]. It is speculated that anatomical changes and, subsequently, distinct vascular configurations may result from a proliferative response involving various vascular wall cells [8]. Our reported case interestingly displayed two of the three vascular abnormalities, more evident on en-face OCT and FA, affecting both significant and smaller vessels, with more pronounced findings in the ischemic right eye.

While retinal vascular abnormalities are relatively common, retinal vascular occlusive disorders are rare [11,12,15-17]. In addition to the intrinsic vascular propensity mentioned earlier, various mechanisms and etiologies have been reported to cause vascular occlusion. These include neurofibromas compressing the vasculature, direct compression from optic nerve gliomas, and retinal toxicity or ischemia resulting from chemotherapy for optic pathway gliomas [11,15-19]. The literature documents retinal ischemia at various levels of occlusion, including the retinal vasculature, ophthalmic artery, and internal carotid artery [11,12,15,16]. Consequently, dedicated vascular imaging, such as arteriography and venography, is crucial. Fortunately, our patient’s results were unremarkable, with no stenosis noted in the carotid or ophthalmic circulations.

Interestingly, despite varying degrees of occlusion being reported, all known cases have involved only one eye [11,12,15-17]. Although the exact cause of this unilateral vascular occlusion remains unknown, it has also been observed in several other organs, including the brain, kidneys, and large vessels of the heart. This phenomenon is a defining characteristic of ischemia in NF1 [15]. In addition to vascular imaging and radiographic assessments, we recommend investigating other potential causes of vascular nonperfusion, such as coagulopathy and inflammatory processes, which were insignificant in our case.

Early detection and treatment of such occlusion are paramount. Multiple reports have highlighted the development of secondary neovascular glaucoma due to ischemic retinas, adding to the risk of visual loss in patients with NF1. Our patient underwent urgent sectoral retinal photocoagulation to the area of retinal nonperfusion, which effectively stabilized her condition, as evidenced by stable follow-up visits [15,16]. Besides photocoagulation, intravitreal anti-vascular endothelial growth factor could further reduce abnormal neovascularization, particularly in the absence of a tractional membrane [16]. However, our patient declined this option after weighing the risks and benefits.

The presentation of our patient was both challenging and intriguing for two reasons. First, she was an 18-year-old with no awareness of her NF1 diagnosis, suggesting that the disease resulted from a de novo mutation. Second, she presented with atypical and rare findings of NF1, specifically vitreous hemorrhage due to retinal neovascularization preceded by retinal ischemia. We believe that reported cases of neovascular glaucoma in NF1 may arise from patients not undergoing routine peripheral fundus assessments to check for retinal ischemia or neovascularization, potentially leading to the development of neovascular glaucoma. Fortunately, our patient did not progress to neovascular glaucoma, which would have significantly impacted her vision had she not received early treatment. Given its significant risk of visual loss, ophthalmologists should always consider retinal ischemia in the context of NF1, even though it is regarded as an atypical association.

## Conclusions

NF1 is an autosomal dominant neurocutaneous disorder diagnosed based on its characteristic clinical findings. The correlation between NF1 and systemic vasculopathy is well established. While it is often considered an uncommon finding, ophthalmic vasculopathy is not an exception and should always be considered. We recommend a thorough fundus examination for all NF1 patients to rule out vascular occlusions. Furthermore, in any patient without additional risk factors, NF1 should be taken into account when making a differential diagnosis of retinal vascular occlusive disease. Awareness of the unique clinical features of NF1 can aid in early diagnosis and improve outcomes through prompt management.
